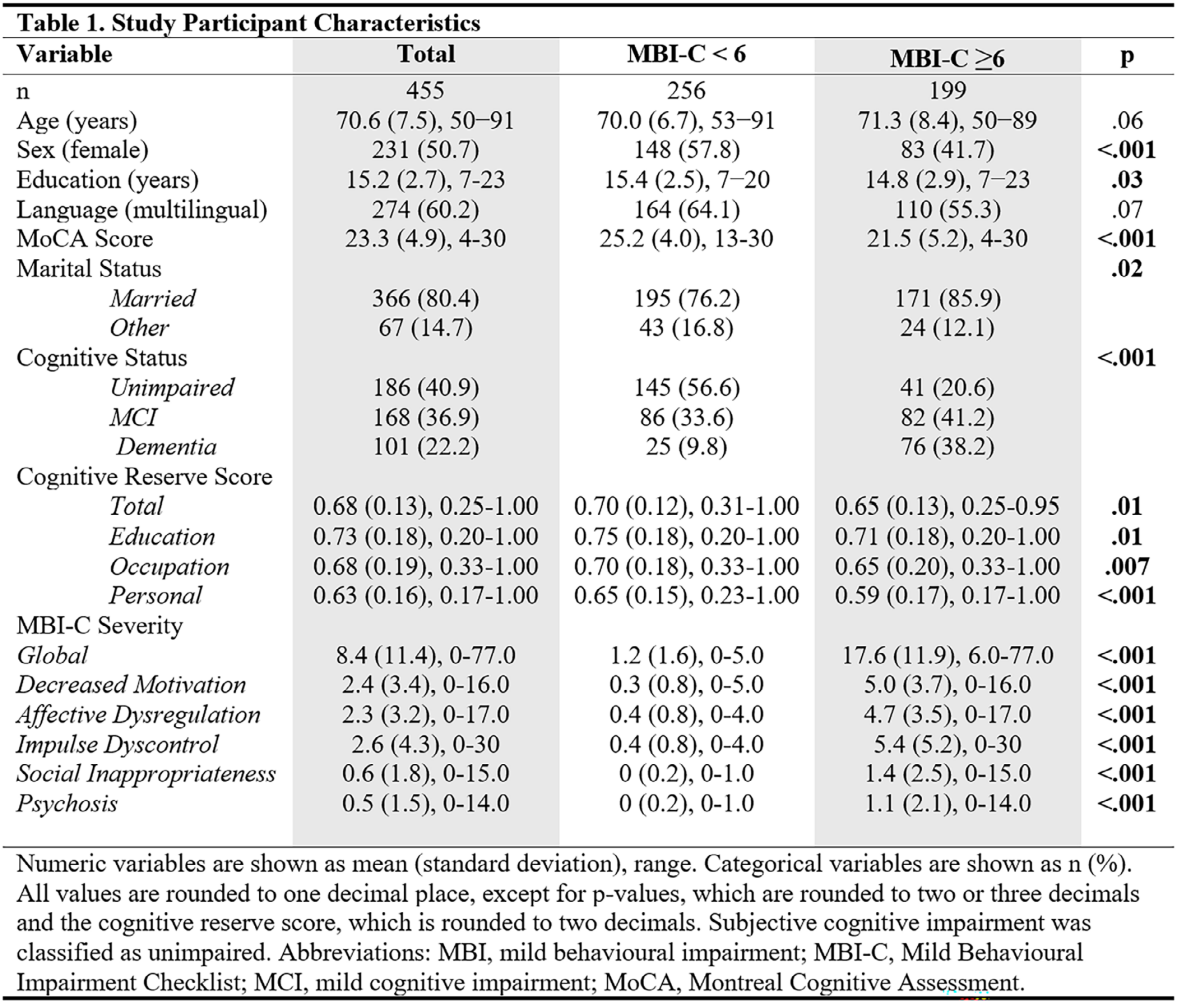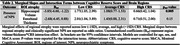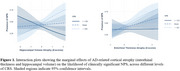# Cognitive reserve as a moderator of the association between hippocampal volume and entorhinal thickness and later‐life behavioral symptoms

**DOI:** 10.1002/alz70857_097686

**Published:** 2025-12-24

**Authors:** Gurshaan Sidhu, Dylan X. Guan, Maryam Ghahremani, Zahinoor Ismail

**Affiliations:** ^1^ University of Calgary, Calgary, AB, Canada; ^2^ Hotchkiss Brain Institute, University of Calgary, Calgary, AB, Canada

## Abstract

**Background:**

Cognitive reserve (CR) can serve as a buffer against the clinical manifestations of neurodegenerative disease pathology. For example, greater CR can mitigate cognitive decline in the presence of Alzheimer disease (AD). However, less is known about the associations between CR and neuropsychiatric symptoms (NPS), which also manifest in neurodegenerative disease. AD‐related cortical atrophy has previously been linked to NPS. Here, we investigated the moderation effect of CR on the relationship between atrophy in the entorhinal cortex and hippocampus, and NPS in older adults.

**Method:**

Data were from 455 participants with normal cognition, mild cognitive impairment, or dementia in the Comprehensive Assessment of Neurodegeneration and Dementia (COMPASS‐ND). Composite CR score (CRS) was derived from established pro‐cognitive proxies across three domains: education, occupation, and personal activities. Convergent validity of the CRS was assessed by modelling association with Montreal Cognitive Assessment (MoCA) score. Logistic regression modelled the relationship between hippocampal/entorhinal atrophy and NPS. A volume/thickness*CRS interaction term was used to assess effect modification, i.e., whether the association between volume/thickness and NPS differed between the levels of CRS. Marginal effects of hippocampal volume and entorhinal thickness were reported across CRS strata (low[‐1SD], average, high[+]). All models adjusted for age and sex; NPS models adjusted for MoCA.

**Result:**

Sample characteristics are shown in Table 1. CRS was associated with higher MoCA score (B=8.48, 95%CI: 5.26‐11.71, *p* <0.001) as well as NPS+ status (OR=0.13, 95%CI: 0.02‐0.65, *p* = 0.01). The association between hippocampal atrophy and NPS+ status was weaker at higher CRS strata (*p* < 0.05), suggesting a protective effect of CR on behaviour. For the association between entorhinal atrophy and NPS+ status, magnitude and direction of the interaction effect were similar to the hippocampus, but the estimate was less precise, and non‐significant [Table 2 for all models].

**Conclusion:**

Findings indicate that greater CR mitigates NPS associated with hippocampal atrophy, independent of cognition, suggesting that CR may provide protective benefits that extend beyond cognition. Future research should explore outcomes longitudinally, consider domain‐specific analyses, and explore whether these findings extend to functional imaging surrogates of CR.